# *In vitro* and *in silico* perspectives on the activation of antioxidant responsive element by citrus-derived flavonoids

**DOI:** 10.3389/fnut.2023.1257172

**Published:** 2023-08-22

**Authors:** Tianzhu Guan, Canfeng Bian, Zheng Ma

**Affiliations:** ^1^School of Food Science and Engineering, Yangzhou University, Yangzhou, China; ^2^Department of Thoracic Surgery, Qilu Hospital of Shandong University, Jinan, China

**Keywords:** dietary antioxidations, citrus-derived flavonoids, oxidative stress, antioxidant, Kelch-like ECH-associated protein 1, structure-activation relationship

## Abstract

**Introduction:**

Oxidative stress plays an essential role in the pathogenesis of chronic diseases. Disrupting the Keap1-Nrf2 pathway by binding Keap1 is identified as a potential strategy to prevent oxidative stress-related chronic diseases. Therefore, of special interest is the utilization of dietary antioxidations from citrus, including narirutin, naringenin, hesperetin, hesperidin, naringin, neohesperidin dihydrochalcone, neohesperidin, and nobiletin, has been exploited as a prospective way to treat or prevent several human pathologies as Keap1-Nrf2 inhibitors for modulation of antioxidant properties.

**Methods:**

To probe into the structural foundation of the molecular identification of citrus-derived antioxidations, we calculated the antioxidant responsive element activation ability of citrus-derived flavonoids after binding with Keap1. Also, the quantum chemistry properties and binding mode were performed theoretically with frontier molecular orbitals, molecular electrostatic potential analysis, molecular docking, and absorption, distribution, metabolism, excretion (ADME) calculation.

**Results and discussion:**

Experimental findings combining computational assays revealed that the tested citrus-derived flavonoids can be grouped into strong agonists and weak agonists. The citrus-derived antioxidations were well housed in the bound zone of Keap1 via stable hydrogen bonding and hydrophobic interaction. Eventually, three of eight antioxidations were identified after ADME and physicochemical evaluations. The citrus-derived flavonoids were identified as potential dietary antioxidants of the Keap1-Nrf2 interaction, and can be used to improve oxidative stress-related chronic diseases.

## 1. Introduction

Recently, growing evidence concerning oxidative stress is broadly perceived as an acute pathological event or a link of an array of chronic inflammatory conditions, such as cardiovascular disease, obesity, diabetes, type 2 diabetes, and Alzheimer’s disease (1, 2). The unbalance between the generation and antioxidant-based degradation of free radicals is triggered by accumulation of reactive oxygen species (ROS) (3, 4). These can modulate essential pathways that significantly affect biofunctions, causing damages to lipids, proteins, and other cellular macromolecules (5–7). Therefore, defense mechanism and sustainable antioxidant have been studied to avoid against numerous forms of oxidative stress. Kelch-like ECH-associated protein 1 (Keap1) and nuclear factor erythroid-2 related factor 2 (Nrf2) are critical bio-macromolecules of the cellular defense system that neutralizes electrophiles and oxidants (8, 9).

Under physiological conditions, the Nrf2 and Keap1 intermolecular interaction is essential in the homeostasis of cellular oxidative stress (10, 11). As the intracellular levels of ROS soar, the Keap1-Nrf2 complex becomes unstable in the cytoplasm. The dissociation of the Keap1-Nrf2 complex initiates the isolation of Nrf2, making reach the nucleus where it acts as a transcriptional stimulator of genes that carry the enhancer sequence antioxidant response element (ARE) (12, 13). Accordingly, a variety of antioxidant enzymes are overexpressed, reducing oxidative stress, and consequently, controlling cell destruction. Hence, the Keap1-Nrf2-ARE system is crucial in oxidation–reduction balance and cell metabolism (14–16).

As a classical pathway against oxidative stress-related chronic diseases, Keap1-Nrf2 pathway modulators are deeply studied as a potential strategy to maintain ROS balance (17). Recent studies have demonstrated the modulatory effect of natural bioactive products via Keap1-Nrf2 signally pathway, such as coumarin-derived compounds, luteolin, curcumin, and diallyl sulfide (18–20). The widely-planted citrus fruits are recognized as highly-consumed fruits in terms of additives, spices, cosmetic components and chemoprophylactic drugs in food, cosmetic and pharmaceutical industries (21–23). The flavonoids derived from citrus fruit extracts possess plentiful bioactive functions, which are regarded as antioxidants against inflammation, allergy, and cancers and lowering blood lipids and cholesterol (24). Therefore, the citrus-derived flavonoids such as narirutin, naringenin, hesperetin, hesperidin, naringin, neohesperidin dihydrochalcone, neohesperidin, and nobiletin, have gained popularity and wide applications.

Due to the Keap1-Nrf2 pathway is a central defensive mechanism against oxidative stress-related chronic diseases, this investigation aimed at synergistically exploring the potential of citrus-derived flavonoids to activate Keap1-Nrf2 signaling via MTT and receptor gene assays. Moreover, the atomistic mechanism about the Nrf2/citrus-derived flavonoids were comprehensively investigated by density functional theory calculation, ADME, and molecular docking. Results obtained from this study may provide new insights into the development of effective Keap1-Nrf2 signaling activators, which may further provide a good resource for discovering preclinical drug candidates for the control and therapy of oxidative stress-associated chronic diseases.

## 2. Materials and methods

### 2.1. Chemicals and reagents

Naringenin, hesperetin, narirutin, naringin, hesperidin, neohesperidin, nobiletin, neohesperidin dihydrochalcone, and *tert*-butylhydroquinone were purchased from Yuanye (Shanghai, China). Dimethyl sulfoxide (DMSO), 3-(4,5-Dimethylthiazol-2-yl)-2,5-diphenyltetrazolium bromide (MTT), and bovine serum albumin (BSA) were purchased from Fluka (Sigma-Aldrich, Shanghai, China). Fetal bovine serum (FBS) and Dulbecco’s modified Eagle medium (DMEM) were provided by Gibco (Grand Island, NY, USA). Lipofectamine 2000 transfection reagent was obtained from Thermo Fisher Science (San Jose, CA, USA). All common reagents used in this study were of analytical grade.

### 2.2. Plasmids

The reporter plasmid (pARE-Luc) was produced by ligating a synthetic double-stranded oligonucleotide tandem ARE target fragment containing the Nrf2 binding site spanning 5’-TGACTCAGC-3′ into a luciferase reporter gene plasmid vector (25). The renilla luciferase was normalized using the control plasmid pRL-SV40 (Promega, WI, USA).

### 2.3. Cell culture and cytotoxicity assay

Human embryonic kidney cell line HEK293T was grown in DMEM enriched with 10% FBS and antibiotics: 100 IU/mL penicillin and 0.1 mg/mL streptomycin. Cells were seeded into 96-well plates and kept in a humidified condition of 5% CO_2_ in a 37°C incubator for 12 h. Cell viability was tested via MTT assay as per the method from (21) with modifications (26). Briefly, after the treatment with the tested chemicals, 10% MTT (5 mg/mL) was added and cultured for 4 h at 37°C. After that, the medium was removed, and the formazan crystals were dissolved in 150 μL of DMSO and then quantified by a SpectraMax i3x microlate reader (Molecular Devices, Sunnyvale, CA, USA) at 570 nm.

### 2.4. Transient transfection and reporter gene assays

According to a previous method by Simmons, reporter gene assays were conducted with minor modifications (27). The 1 × 10^5^ cell/well HEK293T cells were plated at the 24-well plate in phenol red-free DMEM added with 2% dxtran-charcoal plus 10% FBS. According to the manufacturer’s instruction of Lipofectamine 2000, the cells were immediately transfected with plasmids with pARE-Luc and pRL-SV40 after 18 h of incubation. Hereafter, the citrus-derived flavonoids at the level of 10, 20 or 40 μM were added and cultured for 24 h, followed by cell lysis using a passive lysis buffer. Meanwhile, the *tert*-butylhydroquinone, one of the most commonly used phenolic antioxidants, was used as the positive control. Based on the maker’s protocol, both the firefly and Renilla luciferases in cell lysates were tested using a Dual-Luciferase® reporter assay system (Promega, Madison, WI, USA). The luciferase activities were measured using a SpectraMax i3x microlate reader (Molecular Devices).

### 2.5. Density functional investigation

In study of citrus-derived flavonoids, DFT was chosen in all theoretical computations (28, 29). B3LYP hybrid functional is used with the standard 6-311G (d, p) basis set and with ++ diffuse function for the high-quality theoretical geometry optimization via Gaussian 09 (30). For visualization Gauss view 06 is applied. The important molecular orbitals, such as the lowest unoccupied molecular orbital (LUMO), highest occupied molecular orbital (HOMO), and corresponding energy gap, were calculated to clarify the charge transfer possibility within the citrus-derived flavonoids (31). These computational details also provided valuable details on the binding behavior with the receptor.

### 2.6. Molecular electrostatic potential study

To better understanding the chemical reactivity and stability of citrus-derived flavonoids, the molecular electrostatic potential (MSP) and the corresponding quantum descriptors were systematically calculated in detail (32). The visualized demonstrations of MEPs show the sites for electrophilic and nucleophilic location by using Gaussian 09 and Gaussview 06 software. And the auantitative analysis of MSP were performed by multiwfn, which is a powerful program for realizing electronic wavefunction analysis (33).

### 2.7. Molecular docking evaluation

To investigate the binding behavior of the tested chemicals toward Keap1, molecular docking with grid-based energy assessment was performed with the help of docking program Autodock Vina (34). After modification by adding polar H atoms and Kollman charge, the crystal structure of Keap1 in the file 1X2R was used as a docking receptor (35). According to the crystal structure at the receptor binding site, the specific grid box with grid spacing 0.375 Å was generated to enclose all critical residues of Keap1. For each compound, the lowest binding energy (kcal/mol) was calculated based on the Lamarckian genetic algorithm and chosen for final ranking (36, 37). The binding modes between Keap1 and the tested compounds were visualized on PyMol and Discovery studio 2018 (38).

### 2.8. Screening of compounds for drug-likeness and toxicity

Drug-likeness and toxicity can forecast if a specific chemical has properties as an orally active compound (39, 40). To determine the Rule of Five for drug-likeness and toxicity, the canonical simplified molecular input line entry (SMILE) of ligand extracted from PubChem was fed into a freely accessible online tool SwissADME through a login-free website (41).^1^

### 2.9. Statistical analysis

The data were reported as mean ± standard deviation (SD). All assays were carried out independently at least three times, and statistical analysis was conducted using one-way SPSS. All the data were from three independent repeats each treatment (*/#/$, **/##/$$, and ***/###/$$$: *p* < 0.05, *p* < 0.01 and *p* < 0.001).

## 3. Results and discussion

### 3.1. Cytotoxicity of tested compounds in HEK293T cells

**Figure 1:**
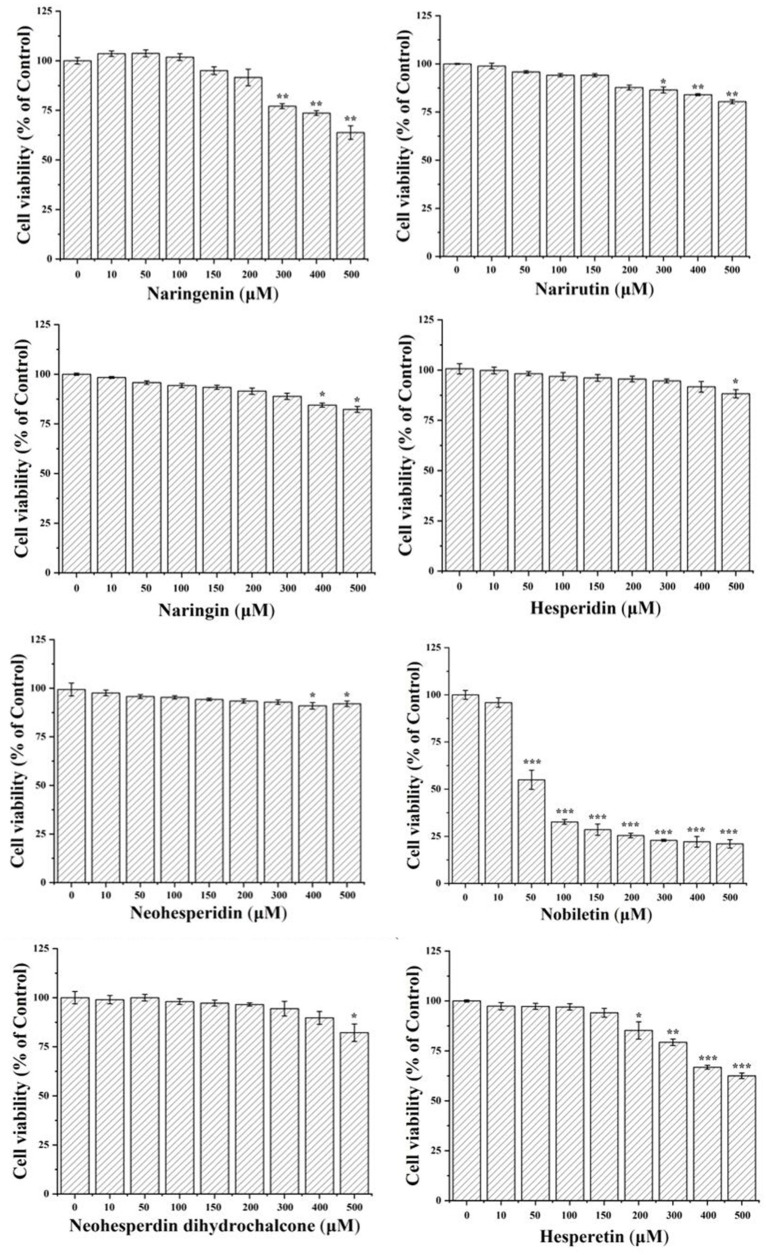
Cell viability of HEK293T cells after treated with citrus-derived flavonoids. All the data were from three independent repeats per treatment and presented as mean ± SD. *, **, and *** represent statistically significant difference of *p* < 0.05, *p* < 0.01, and *p* < 0.001, respectively.

In order to performed the luciferase reporter gene assays, the concentration range of citrus-derived flavonoids with no significant inhibited effect of HEK293T should be determined first. This confirmed the changes in ARE activation activity were due to xenobiotic compound exposure, rather than the side effect of cell death. The tested compounds at a concentration of 50 μM did not exhibit cytotoxic effect (Figure 1), therefore, 10, 20, and 40 μM were chosen for the subsequent experiments.

### 3.2. Citrus-derived flavonoids induced are activation in a dose-dependent manner

**Figure 2:**
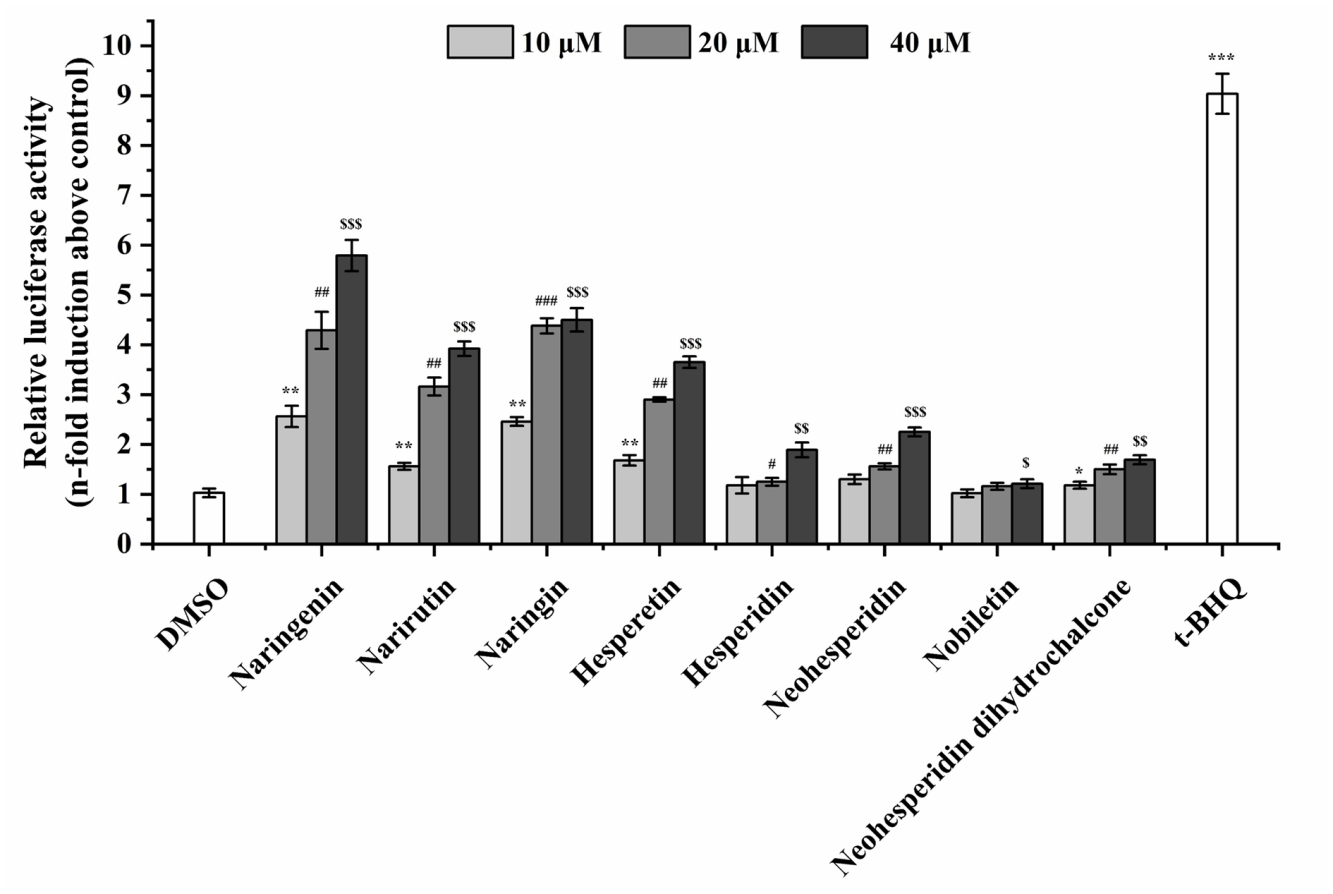
The citrus-derived flavonoids activated transcription of ARE. HEK293T cells were co-transfected with pARE-Luc and pRL-SV40 by lipofection, and then treated with flavonoids or *tert*-butylhydroquinone (*t*-BHQ) for 24 h. Results are given as means ± SD of three independent experiments. All the data were from three independent repeats per treatment and presented as mean ± SD. */#/$, **/##/$$, and ***/###/$$$ represent statistically significant difference of *p* < 0.05, *p* < 0.01, and *p* < 0.001, respectively.

To assess the ARE-driven luciferase activity of citrus-derived flavonoids, ARE-driven reporter gene assay *in vitro* was constructed. The luciferase activity of the transient transfection system in HEK293T cells was measured after 24 h of incubation. The luciferase activity of HEK293T cells was significantly induced by 12.5 μM *tert*-butylhydroquinone, as shown in Figure 2. The citrus-derived flavonoids also induced transactivation of ARE in a dose-dependent manner. Two categories were classified: strong agonists (naringenin, narirutin, naringin, hesperetin) and weak agonists (hesperidin, neohesperidin, nobiletin, neohesperidin dihydrochalcone).

### 3.3. The FMOs study of citrus-derived flavonoids

**Figure 3:**
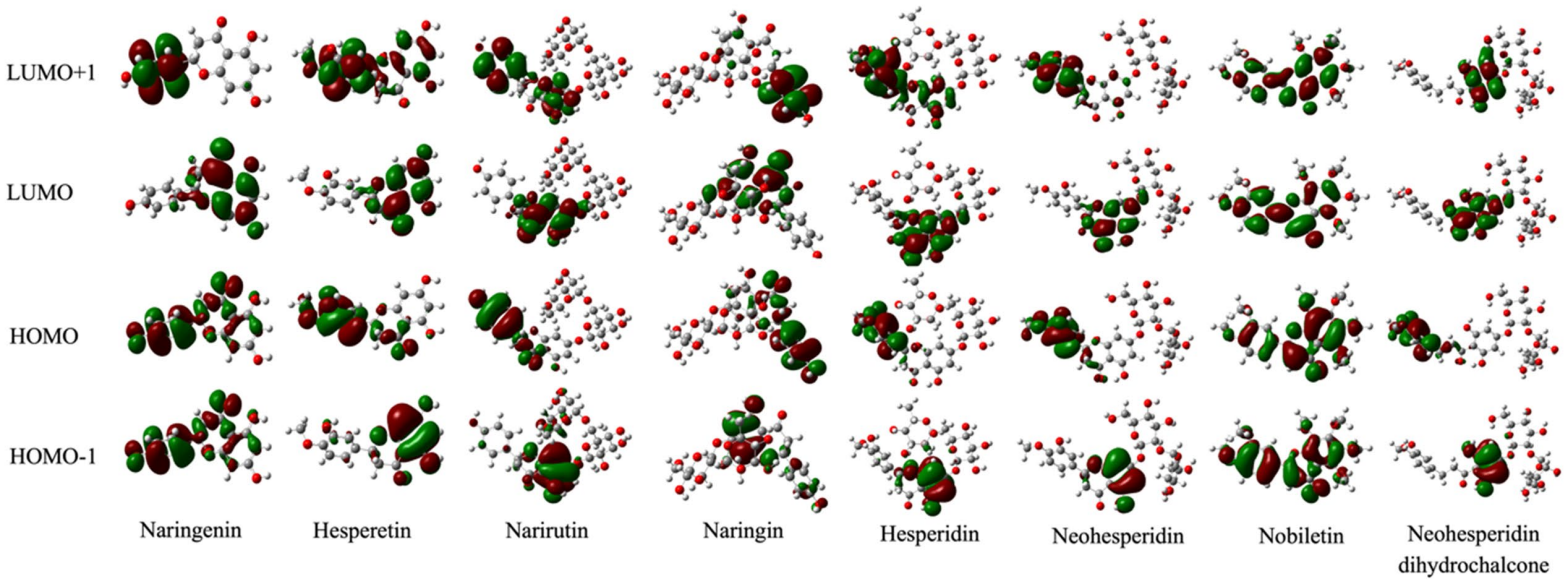
The molecular orbitals for the HOMO-1, HOMO, LUMO, and LUMO+1 of citrus-derived flavonoids.

As a reliable approach for related organic molecules, DFT/B3LYP hybrid functional with 6-311G (d, p) was broadly applied to calculate and analyze the quantum chemistry parameters in this study (42, 43). Specifically, LUMO+1, LUMO, HOMO, and HOMO-1, which collectively called FMOs, play a crucial role in the chemical characteristics of the bioactive compounds (44, 45). Among them, the HOMOs and LUMOs represent the electron-providing and -accepting abilities of the tested chemicals (46). The three-dimensional plots and energy results of FMOs were tabulated in Figure 3 and Table 1. The negative and positive phases were represented in green and red, respectively. The computations found that the HOMOs and LUMOs of nobiletin were mainly localized over the entire molecule. The LUMO+1, LUMO, HOMO, and HOMO-1 energies are −0.02560, −0.6004, −0.21727, and − 0.22707, respectively. While the HOMO of naringenin is positioned on nearly the whole structure, its LUMO is particularly located on the phenol group. The energy gap represents the chemical reactivity and intramolecular charge of the compounds. A larger energy gap means lower kinetic stability, and vice versa. The DFT calculated energy gaps of naringenin, hesperetin, naringin, and narirutin were 0.1794, 0.17517, 0.17388, and 0.16772 eV respectively, which are associated with the decreasing energy gap and stand for the reactivity of the structures.

**Table 1:** Calculated energies values of citrus-derived flavonoids.

Compounds	E_LUMO + 1_ (eV)	E_LUMO_ (eV)	E_HOMO_ (eV)	E_HOMO-1_ (eV)	E _HOMO-LUMO_ Gap (eV)
Naringenin	−0.00575	−0.03810	−0.21750	−0.22379	0.17940
Hesperetin	−0.00356	−0.04036	−0.21553	−0.22615	0.17517
Narirutin	−0.01479	−0.05822	−0.22594	−0.23922	0.16772
Naringin	−0.00358	−0.04234	−0.21622	−0.22768	0.17388
Hesperidin	0.00726	−0.05032	−0.19922	−0.21628	0.14890
Neohesperidin	0.00176	−0.05224	−0.20745	−0.21611	0.15521
Nobiletin	−0.02560	−0.6004	−0.21727	−0.22707	−0.38313
Neohesperidin dihydrochalcone	0.00491	−0.05191	−0.20126	−0.22530	0.14935

### 3.4. The topology properties of citrus-derived flavonoids

**Figure 4:**
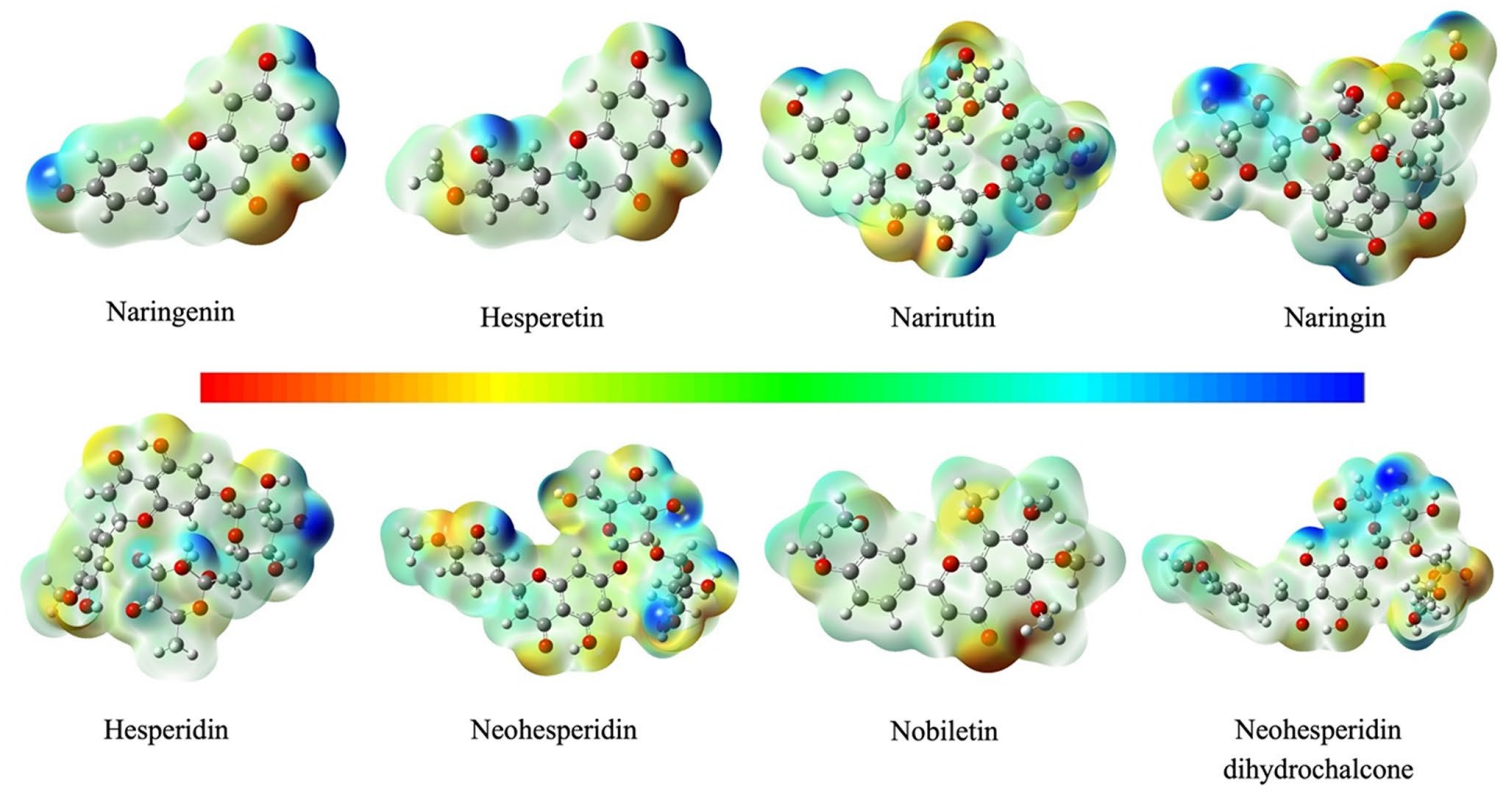
The molecular electrostatic potential (MEP) of citrus-derived flavonoids.

Based on the DFT computation at the same level of theory, the MEP of chemicals could visually represent the electronic distribution (47). The calculated MEPs in Figure 4 show a surface analysis throughout the tested compounds calculated with the B3LYP density functional method. Potential ranks as red < green < blue. The red region represents negative electrostatic potential, and indicate the proton is attracted by the aggregate electron density in the molecule and related to electrophilic reactivity. MEP surface plots have been traced with the help of the different colors as the given significance of electrostatic: the blue regions are the positive electrostatic potential, and suggest the proton is repelled by the atomic nuclei and associated with nucleophilic reactivity. The most electrophilic attacks occur on the C=O (Figure 4). Moreover, the blue-green region corresponds to the hydroxyl of benzene ring in the tested chemicals. The MEP results imply that the hydrogen and oxygen atoms suggest the strongest attraction and repulsion, respectively, which may affect the electrostatic interactions between ligands and surrounding residues of Keap1 and subsequently influence their binding potency.

Since the electrostatic potentials greatly account for the interaction behavior between the receptor and ligands, quantitative study of molecular surface is necessary. The global minimum on the surface of the tested compound occurred at C=O nearly −50 kcal/mol, which is the largest minimum value on the surface (Table 2). The global minimum arising from the cationic hydroxyl varies largely from 302.19627 to 671.84562 kcal/mol. In comparison, more electronegative O atoms connected to the H atoms attract the electron cloud, making the H atoms more positive. The internal charge separations of strong agonists are generally higher than weak agonist groups.

**Table 2:** Quantitative analysis of molecular electrostatic potential.

Parameters	Naringenin	Narirutin	Naringin	Hesperetin	Hesperidin	Neohesperidin	Nobiletin	Neohesperidin dihydrochalcone
Volume (Angstrom^3)	302.19627	623.87043	621.56982	335.10534	653.45142	658.60285	462.35190	671.84562
Minimal value (kcal/mol)	−52.59893	−49.79904	−49.56763	−51.60869	−45.25282	−42.47707	−48.01324	−50.35080
Maximal value(kcal/mol)	60.13802	61.43728	58.59879	61.29152	60.79677	51.60445	17.04324	59.52240
Overall surface area (Angstrom^2)	283.86467	507.81990	494.37966	313.01741	528.94389	560.95240	421.43319	575.99629
Positive surface area (Angstrom^2)	128.01047	291.84662	268.26315	149.37680	274.92109	268.65558	228.58465	299.82638
Negative surface area (Angstrom^2)	155.85420	215.97328	226.11651	163.64061	254.02280	292.29682	192.84854	276.16991
Overall average value (kcal/mol)	−0.44340	1.97470	1.53791	−0.29428	0.73501	−0.30582	−1.09868	1.31316
Positive average value (kcal/mol)	15.43217	14.46651	14.74237	13.98182	13.38476	13.92216	7.35345	14.01082
Negative average value (kcal/mol)	−13.48275	−14.90560	−14.12777	−13.32599	−12.95543	−13.38302	−11.11704	−12.47216
Balance of charges (nu)	0.23780	0.24993	0.24909	0.24070	0.24039	0.24659	0.11022	0.24468
Internal charge separation (kcal/mol)	14.32292	14.45547	14.38153	13.62801	13.16245	13.63018	9.20900	13.26088
Molecular polarity index (kcal/mol)	14.36185	14.65326	14.46127	13.63896	13.17858	13.64123	9.07568	13.27309

### 3.5. Binding mode of citrus-derived flavonoids with Keap1

**Figure 5:**
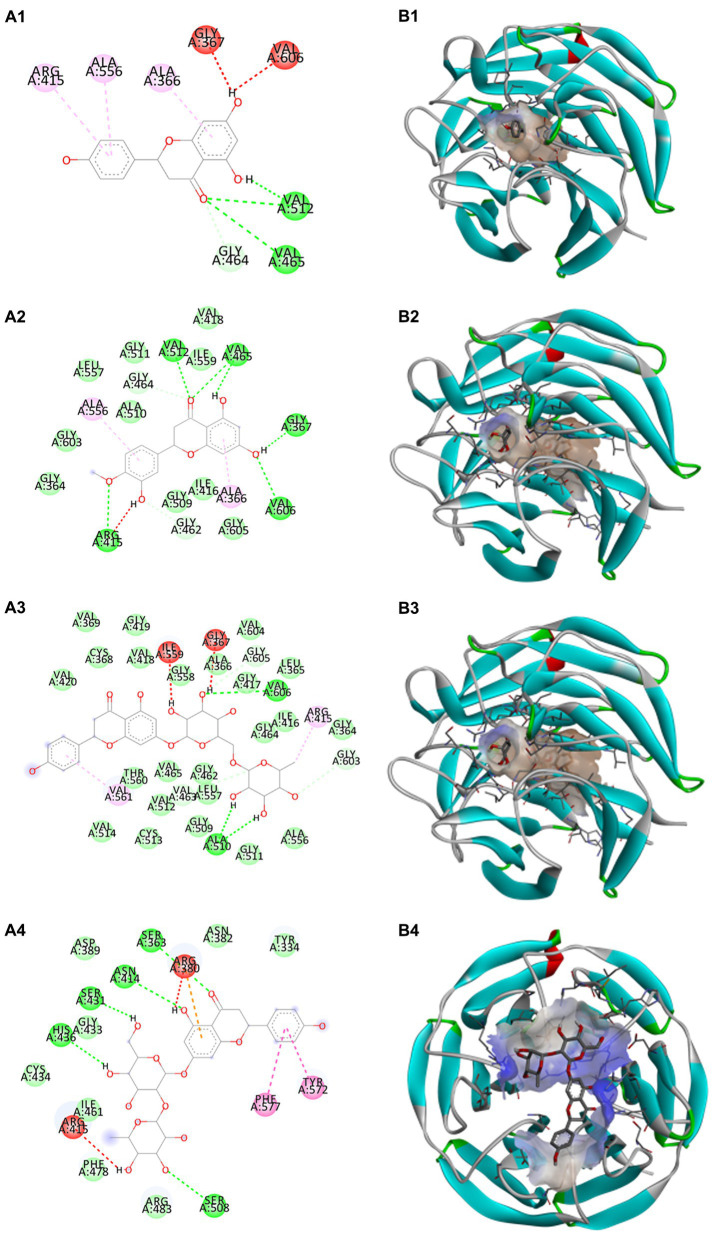
Structural representation of the keap1-ligand complex with strong agonist mode. **(A1–A4)** are the 2D representations of naringenin, hesperetin, narirutin, and naringin. **(B1–B4)** are the 3D representations of naringenin, hesperetin, narirutin, and naringin.

**Figure 6:**
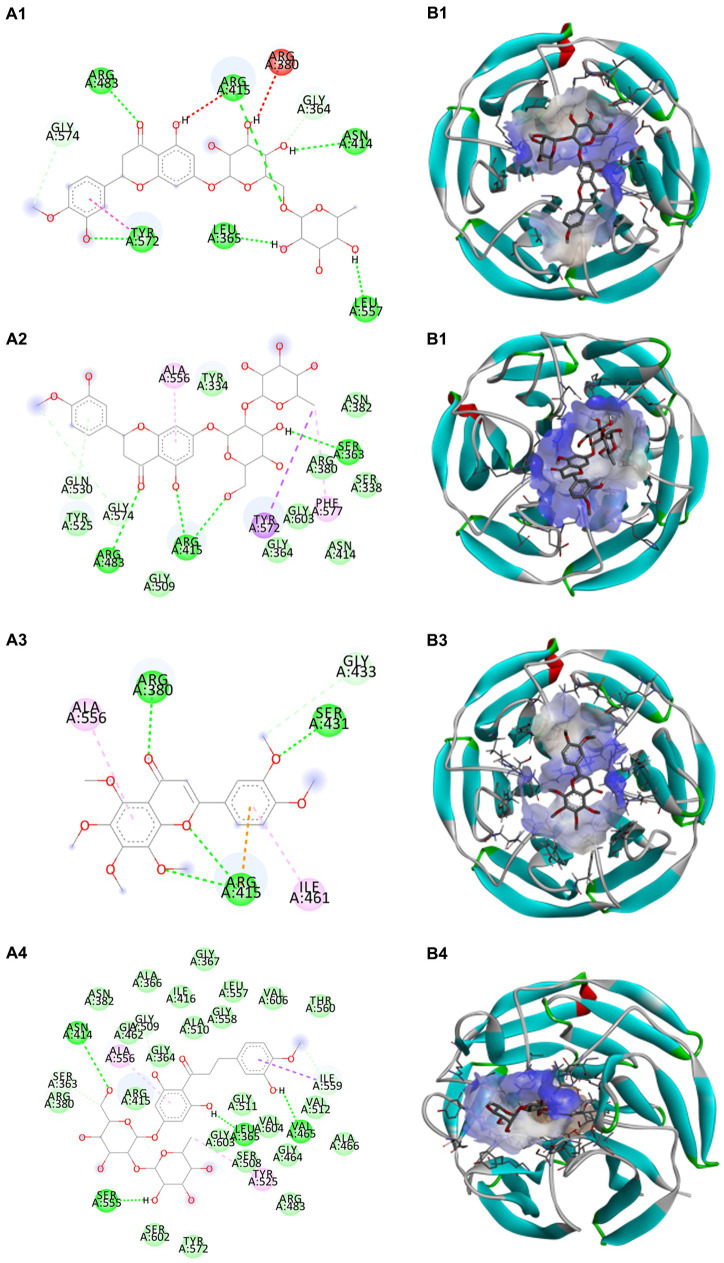
Structural representation of the keap1-ligand complex with weak agonist mode. **(A1–A4)** are the 2D representations of hesperidin, neohesperidin, nobiletin, and neohesperidin dihydrochalcone. **(B1–B4)** are the 3D representations of hesperidin, neohesperidin, nobiletin, and neohesperidin dihydrochalcone.

Docking calculations between the compounds and Nrf2 reproduced the tested binding conformations, as found in Keap1-Nrf2 complex (PDB: 1X2R), implying the Keap1 model is feasible for calculating binding free energy. Docking calculations showed the binding affinity of the tested compounds with Keap1 ranked as narirutin > naringenin > hesperetin > hesperidin > naringin = neohesperidin dihydrochalcone > neohesperidin > nobiletin (Table 3). In the 2D and 3D representations in Figures 5, 6, the dark green, light green orange, pink, red, and cyan represent hydrogen bond, van der Waals, carbon hydrogen bond, and Pi-cation, respectively (35). Based on the experiments above, these compounds can be divided into weak agonists (naringin, neohesperidin dihydrochalcone, neohesperidin, nobiletin) and strong agonists (narirutin, naringenin, hesperetin, hesperidin). Basically, the order of activation potency for compounds agrees well with the binding affinity with Keap1. The docking results showed the top three molecules (naringenin, hesperetin, narirutin) were well accommodated in the binding pocket of Keap1 with high hydrophobicity. Except the two unfavorable donor-donor interactions, the hydroxyl groups and carbonyl of naringenin form three hydrogen bonds with VAL^512^ and VAL^465^, which contribute to the high binding energy between Keap1 and naringenin. Similarly, in addition to the three hydrogen bonds with VAL^512^ and VAL^465^, the hydroxyl groups of hesperetin also form other hydrogen bonds with GLY^367^, VAL^606^, and ARG^415^. Different from naringin, narirutin enters the binding pocket of Keap1 with the highest binding affinity and exhibits different interactions via hydrogen bonds, van der Waals, and Pi-Alkyl interactions (48). Additionally, the binding mode of weak agonists is strikingly different from that of the strong agonists. The weak agonists form several hydrogen bonds with the surrounding residues of Keap1, such as ARG^380^, SER^431,^ ARG^483^, ASN^414^, ARG^415^, LEU^365^, and TYR^572.^ The multiple hydrogen bonds as well as Pi-Alkyl and Pi-Sigma interactions account for the stability and closeness between the ligand and Keap1.

**Table 3:** Structural representations of keap1-ligand complex.

Compounds	Chemical structures	Affinity (kcal/mol)	Agonist potency
Naringenin	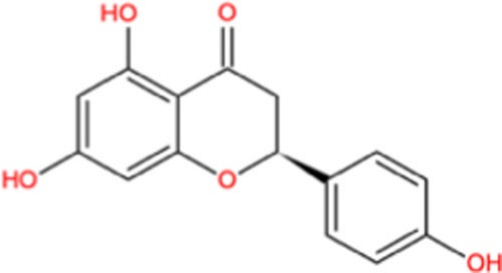	−9.9	Strong agonist
Hesperetin	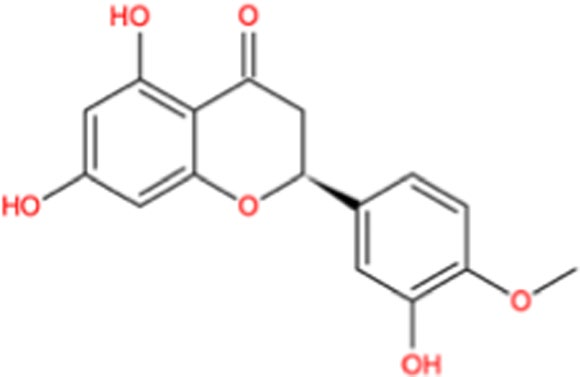	−9.7	Strong agonist
Narirutin	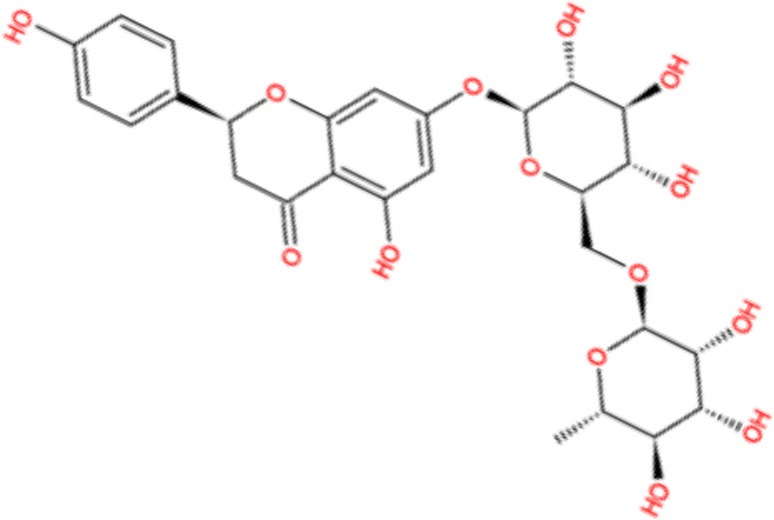	−10.7	Strong agonist
Naringin	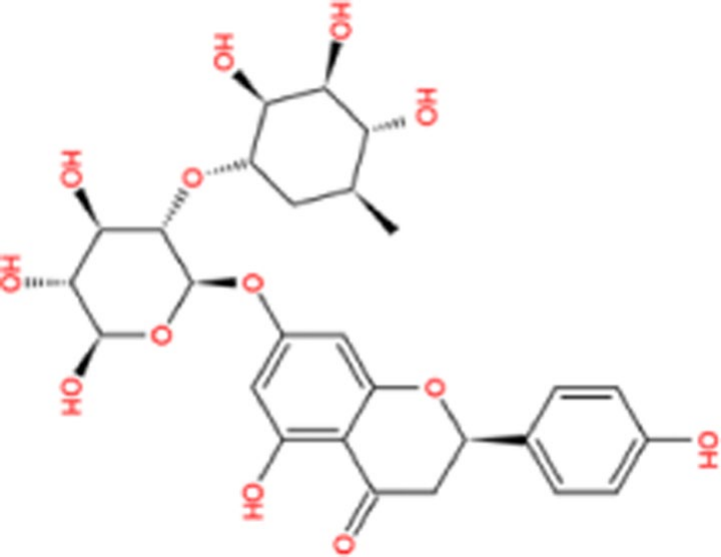	−8.8	Strong agonist
Hesperidin	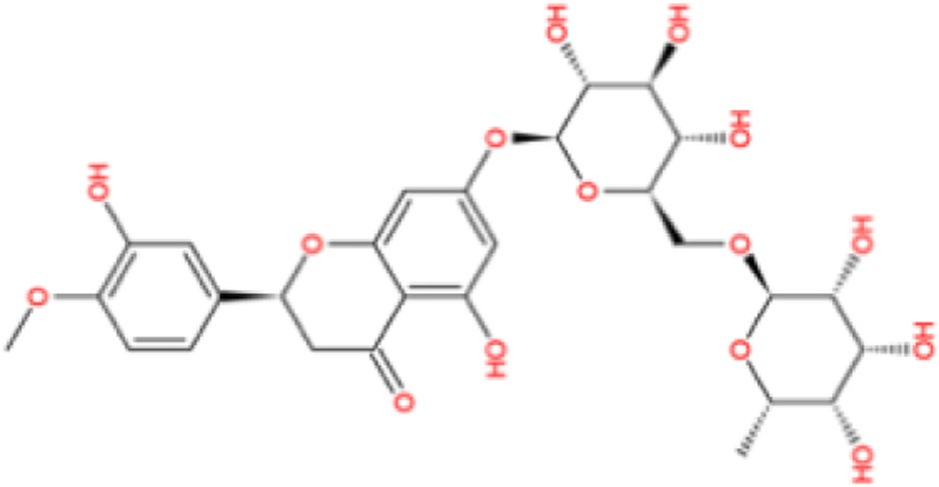	−9.6	Weak agonist
Neohesperidin	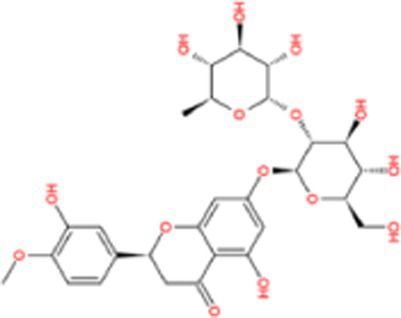	−8.3	Weak agonist
Nobiletin	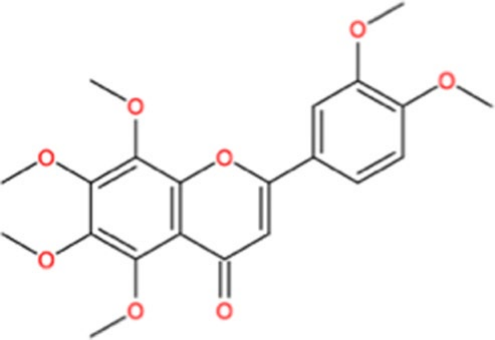	−6.7	Weak agonist
Neohesperidin dihydrochalcone	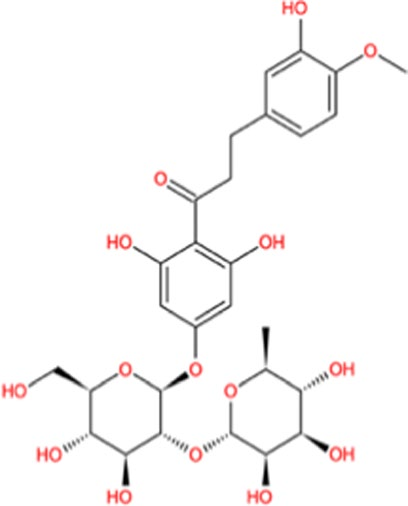	−8.8	Weak agonist

### 3.6. *In silico* pharmacokinetics properties

With the rapid increase in biological and chemical information, evaluation of physicochemical and ADME properties is vital in discovery of phytocompounds from traditional plants. To detect bioavailability potential of phytocompounds, the pharmacokinetics, bioavailability, drug-likeness and medicinal chemistry friendliness of the citrus-derived flavonoids were characterized primarily based on “Lipinski’s rule of five,” which are the molecular mass shall not surpass 500 Dalton, there shall not be over five hydrogen bond donors or over ten acceptors, and the partition index LogP shall not exceed 5. The Swiss ADME results (Table 4) show that naringenin, hesperetin, and Nobiletin satisfy Lipinski’s rule of five with zero violations. Others showing a violation of more than two rules depict a molecule as not orally active. Hence, the three tested compounds can be candidates for further studies.

**Table 4:** Drug-likeness predictions of compounds computed by SwissADME.

Compounds	Molecular weight	Number of H-bond acceptor	Number of H-bond donor	Number of rotatable bonds	LogP
Naringenin	272.25	5	3	1	1.75
Narirutin	580.53	14	8	6	2.13
Naringin	580.53	14	8	6	2.38
Hesperetin	302.28	6	3	2	2.24
Hesperidin	610.56	15	8	7	2.60
Neohesperidin	610.56	15	8	7	2.57
Nobiletin	402.39	8	0	7	3.96
Neohesperidin dihydrochalcone	612.58	15	9	10	2.29

The predictive data of individual ADME behaviors of the tested compounds were summarized in Table 5, such as water solubility, pharmacokinetics, bioavailability, drug-likeness, and medicinal chemistry. The skin absorption of the tested chemicals was reflected by skin permeability (Kp, cm/s). A more negative logKp means less skin absorption. The Kp of all the compounds is within −6.17 and −10.54, which presumes low skin permeability. Additionally, layout and absorption of drug molecules were detected using blood brain barrier (BBB) and gastrointestinal (GI) permeation. The SwissADME forecasting variables show all compounds have GI absorption, especially naringenin, hesperetin, and nobiletin. Hence, ADME prediction and physicochemical analysis suggest naringenin, hesperetin, and nobiletin may be better candidates than other tested chemicals.

**Table 5:** ADME predictions of citrus-derived flavonoids.

Compounds	Water solubility		Pharmacokinetics			Druglikeness				Medicinal chemistry	
	Log S (ESOL)	Log S (Ali)	GI absorption	BBB permeant	Log K_p_	Lipinski	Ghose	Muegge	Bioavailability Score	PAINS	Leadikeness
Naringenin	−3.49 Soluble	−3.99 Soluble	High	No	−6.17	Yes	Yes	Yes	0.55	0 alert	Yes
Narirutin	−2.64 Soluble	−3.25 Soluble	Low	No	−10.54	No	No	No	0.17	0 alert	No
Naringin	−2.98 Soluble	−3.82 Soluble	Low	No	−10.15	No	No	No	0.17	0 alert	No
Hesperetin	−3.62 Soluble	−4.27 Moderately Soluble	High	No	−6.30	Yes	Yes	Yes	0.55	0 alert	Yes
Hesperidin	−3.28 Soluble	−4.33 Moderately Soluble	Low	No	−10.12	No	No	No	0.17	0 alert	No
Neohesperidin	−3.07 Soluble	−3.98 Soluble	Low	No	−10.36	No	No	No	0.17	0 alert	No
Nobiletin	−4.18 Moderately Soluble	−4.47 Moderately Soluble	High	No	−6.62	Yes	Yes	Yes	0.55	0 alert	No
Neohesperidin dihydrochalcone	−3.00 Soluble	−4.39 Moderately Soluble	Low	No	−10.25	No	No	No	0.17	0 alert	No

## 4. Discussion

The imbalance between excessive generation of ROS and the antioxidant enzyme system of oxidative stress may lead to several acute injury (44). Recognizing the role of oxidative stress results in the suggested utilization of plant-sourced antioxidants for the prevention and control of oxidative stress-related damages (49). Thus, suppressing oxidative stress by plant-derived compounds may assume a potential way to alleviate the development and occurrence of various diseases (44). Citrus-derived flavonoids (e.g., narirutin, naringenin, hesperetin, hesperidin, naringin, neohesperidin dihydrochalcone, neohesperidin, nobiletin) are rich in citrus fruits and are comparable in molecular structures with the acknowledged Nrf2 agonist. Therefore, these natural compounds may potentially act as agonists in the Keap1-Nrf2-ARE antioxidant pathway. To investigate the anti-oxidative stress behavior of the tested compounds, we transiently co-transfected HEK293T cells with the plasmids of pARE-Luc and pRL-SV40 and thereby explored the activation effect of the tested chemicals on the Keap1-Nrf2-ARE pathway. The transfection efficiency was about 50–60% and the transfected cells were processed with the tested compounds (10, 20, and 40 μM). All of the eight compounds presented different levels of ARE activation activity (Figure 2), which may highlight the potential of the tested citrus-derived flavonoids in promoting Nrf2-ARE connection and the ARE-induced target gene expression.

To establish the follow-up prediction modeling, our main effort was to investigate the relationships between chemical reactivity and ARE activation of the chemicals. Thus, based on experimental and computational studies, the descriptors of the Keap1 and citrus-derived flavonoids interaction may be a novel way to bridge the activation ability *in vitro* and binding prediction *in vivo*. To identify more detail, the analysis of FMOs presents details on the HOMO to LUMO electron migration, which describes chemical species reactivity. The corresponding energy was executed for all the tested compounds using the quantum mechanical DFT methodology. The HOMO is mainly positioned on the ring C and N atoms while the LUMO exists along the C and O atoms (Figure 3). The energies of four major molecular orbitals of the compounds *viz.* the HOMO and the second HOMO (HOMO-1), the LUMO and the second LUMO (LUMO+1) were determined using B3LYP/6–311++G (d, p) (Table 1). For the strong agonist naringenin, the band gap energy of the molecule computed from HOMO (−0.21750 eV) and LUMO (−0.03810 eV) was 0.17940 eV, which is similar to that of bioactive molecules. In comparison, Nobiletin has the lowest energy gap of −0.38313 eV with HOMO of −0.6004 eV and LUMO of −0.21727 eV. To quantitatively evaluate the molecular polarity based on the MEP distribution, the results of molecular polarity index were presented in Table 2. Basically, group I (Naringenin, Narirutin, Naringin, Hesperetin) were significantly higher than group II (Hesperidin, Neohesperidin, Nobiletin, Neohesperidin dihydrochalcone), which indicating the obvious polarity of the group I and might influence the potential binding activity of receptor-ligand complex. It is worth noting that the properties of charge separation were also obtained from the optimized structures of citrus-derived flavonoids. In accordance with molecular polarity index, group I has higher degree of charge separation than group II.

Thus, in-depth molecular docking helps uncover the ligand-Keap1 binding and assess the potential prevention impact in the *in-vivo* system (50). Noteworthily, in the classic activation of the Keap1/Nrf2 pathway, agonist induces transient modifications in Keap1 by interacting with specific residues (48, 51). For instance, naringenin makes two hydrogen bonds interact with VAL^512^ and VAL^465^; it also hydrophobically interacts with GLY^464^ in the binding site of Keap1. Nobiletin, as a weak agonist, acts in a multi-hydrogen-bond pattern with ARG^380^, SER^431^, and ARG^415^ residues in the binding pocket. It also forms π-π interactivity with ALA^556^ and ILE^461^ of Keap1.

## 5. Conclusion

Summing up the above, we evaluated the cytotoxicity and ARE activation of citrus-derived flavonoids using MTT and receptor gene assays. All the tested compounds showed agonist activity at different concentrations. Computational approaches such as quantum calculation and molecular docking were used to systematically evaluate the Keap1-ligands interactions. The MSP and FMOs results showed the H atoms and N atoms with the strongest attraction and repulsion, respectively, may affect the electrostatic interactions between citrus-derived flavonoids and Keap1, and subsequently influence the binding potency. Quantitative analysis of MEP is an extraordinary technique for analyzing non-covalent interaction, and predicting the reactive sites and reactivity of the tested compounds. After calculation on swissADME, selection of the eight citrus-derived flavonoids showed that naringenin, hesperetin, and nobiletin may be prospective pharmacological candidates for further drug making owing to their drug-likeness and ADME properties. This selection is also consistent with the activation ability results. In summary, the tested compounds are identified as potential dietary antioxidants of Keap1-Nrf2 interaction, which can be used for the control and therapy of oxidative stress-associated chronic diseases.

## Data availability statement

The original contributions presented in the study are included in the article/supplementary material, further inquiries can be directed to the corresponding author.

## Ethics statement

Ethical approval was not required for the studies on animals in accordance with the local legislation and institutional requirements because only commercially available established cell lines were used.

## Author contributions

TG: Software, Writing – original draft, Funding acquisition. CB: Data curation, Formal analysis, Writing – original draft. ZM: Validation, Visualization, Writing – review and editing.

## Funding

The author(s) declare financial support was received for the research, authorship, and/or publication of this article.

This work was supported by the Jiangsu Provincial Research Foundation for Basic Research of China (Grant No. BK20200954) and the National Youth Talent Support Program of the China Association for Science and Technology (20220615ZZ07110388).

## Conflict of interest

The authors declare that the research was conducted in the absence of any commercial or financial relationships that could be construed as a potential conflict of interest.

## Publisher’s note

All claims expressed in this article are solely those of the authors and do not necessarily represent those of their affiliated organizations, or those of the publisher, the editors and the reviewers. Any product that may be evaluated in this article, or claim that may be made by its manufacturer, is not guaranteed or endorsed by the publisher.

## Footnotes

^1^ www.swissadme.ch/
